# Examined lymph node count is not associated with prognosis in elderly patients with pN0 thoracic esophageal cancer

**DOI:** 10.1097/MD.0000000000024100

**Published:** 2021-01-15

**Authors:** Guoqing Zhang, Xiaofeng Guo, Lulu Yuan, Zhen Gao, Jindong Li, Xiangnan Li

**Affiliations:** a Department of Thoracic Surgery, First Affiliated Hospital of Zhengzhou University, Zhengzhou; b Department of Thoracic Surgery, Anyang Tumor Hospital, Anyang, Henan Province, China.

**Keywords:** esophageal cancer, esophagectomy, elderly, surveillance, epidemiology, end results (SEER)

## Abstract

The purpose of this study was to determine whether the number of lymph nodes dissected predicts prognosis in surgically treated elderly patients with pN0 thoracic esophageal cancer. We searched the Surveillance, Epidemiology, and End Results database and identified the records of younger (<75 years) and older (≥75 years) patients with pN0 thoracic esophageal cancer between 1998 and 2015. The patient characteristics, tumor data, and postoperative variables were analyzed in this study. The Kaplan-Meier method and a Cox proportional hazard model were used to compare overall and cause-specific survival. Data from 1,792 esophageal cancer patients (older: n = 295; younger: n = 1497) were included. The survival analysis showed that the overall and cause-specific survival in the patients with ≥15 examined lymph nodes (eLNs) was significantly superior to that in the patients with 1 to 14 eLNs (*P* < .001); however, the difference disappeared in the older patients. After stratification by the tumor location, histology, pT classification, and differentiation between the younger and older cohorts to analyze the association between eLNs and survival, we found that the differences remained significant in most subgroups in the younger cohort. There were no differences in any subgroups of older patients. This study replicated the previously identified finding that long-term survival in patients with extensive lymphadenectomy was significantly superior to that in patients with less extensive lymphadenectomy. However, less extensive lymphadenectomy may be an acceptable treatment modality for elderly patients with pN0 thoracic esophageal cancer.

## 1 Introduction

Currently, esophagectomy with lymphadenectomy is the mainstay therapy for esophageal cancer without systemic metastases, and the lymph node (LN) status is regarded as a valid risk stratification tool that significantly influences the recurrence rates and survival in patients undergoing surgery. Despite the prognostic significance of the identification of LN metastases in patients with esophageal cancer, the optimal number of examined lymph nodes (eLNs) needed for an esophagectomy is controversial and requires clarification.^[3,4,16,18]^

In the past decade, many studies that examined the impact of eLNs on the survival of patients with esophageal cancer have shown that there was a significant improvement in prognosis with an increasing number of nodes examined,^[2,10–12,18,19]^ even in patients with pathologic N0 disease.^[2,12,19,20]^ Additionally, the reasonable cutoff value for the adequate staging of esophageal carcinoma remains controversial as a result of the different numbers of cases, different inclusion criteria, and different statistical methods used. Currently, the American Joint Committee on Cancer (AJCC) staging system for esophageal cancer recommends that 10 nodes must be resected to improve the N categorization and obtain a therapeutic effect of lymphadenectomy. However, the National Comprehensive Cancer Network recommends sampling at least 15 LNs at the time of esophagectomy. Indeed, examining more LNs may eliminate micrometastatic lymph nodes, increase the likelihood of accurate staging and influence survival. However, more extensive lymphadenectomy is a highly invasive procedure that can lead to an increased risk of complications, prolonged hospitalization, or increased mortality. Because of the current relatively higher life expectancy, the number of elderly patients with esophageal cancer has significantly grown in recent decades,^[8]^ and approximately 29.4% of patients are aged ≥75 years (defined as elderly individuals by the American Geriatrics Society^[9]^) at the time of diagnosis. It is well known that the elderly population is generally characterized as having more comorbid illnesses and reduced normal organ function. The deterioration in the physical condition resulting from esophagectomy may be severe in elderly patients, and an advanced age may negatively affect prognosis.^[14,21]^ However, knowledge regarding the association between eLNs and prognosis in elderly patients is limited.

In the present study, we used a population-based cancer registry to compare the effect of the eLN count on prognosis in elderly patients with pN0 thoracic esophageal cancer.

## 2 Materials and methods

### 2.1 Data source and case selection

The Surveillance, Epidemiology, and End Results (SEER) database, which originates from 18 cancer registries covering almost 28% of the United States population, collects and publishes cancer incidence and survival data. Esophageal cancer cases (site codes, C15.0–C15.9) from 1973 to 2015 were identified in the SEER database. Eligible patients were identified as those with pathologically confirmed esophageal squamous cell carcinoma or adenocarcinoma located in the thoracic esophagus (pathologic T1-3N0M0) as defined by the International Classification of Diseases for Oncology (ICD-O-3)/World Health Organization 2008 site code C153 to C155 who underwent esophagectomy (codes, 30–80). Patients diagnosed before 1998 were excluded because there was no specific AJCC 6th to 7th edition staging information prior to 1998. Patients without detailed information regarding their race, marital status, grade, pathologic T classification (pT classification), eLNs, and survival months and patients with zero eLNs or a history of other malignant diseases or other concurrent malignant diseases of the esophagus and other organs were excluded. We also excluded patients who underwent neoadjuvant therapy, which has been associated with fewer eLNs.^[12]^

This study was deemed exempt from review by the Zhengzhou University Institutional Review Boards.

### 2.2 Statistical analysis

The descriptive statistics of the continuous variables are expressed as the means ± standard error. Independent sample t-tests were used to compare the normally distributed continuous variables. *χ*^2^ tests were used to compare the categorical variables. Multivariate Cox proportional hazards analyses were used to identify the variables independently associated with overall and cause-specific survival. The variables entered into the model included the patients’ characteristics (age at diagnosis, year of diagnosis, sex, race, and marital status), tumor data (tumor location, histology, pT classification, eLNs, SEER historic stage, and grade), and postoperative variables (survival months). Overall and cause-specific survival were analyzed using a Kaplan-Meier analysis and log-rank test. All statistical analyses were performed using SPSS version 22 for Windows (SPSS Inc., Chicago, IL). The statistically significant differences were determined using a 2-tailed test, and *P*-values less than .05 were considered statistically significant.

## 3 Results

### 3.1 Patient characteristics

In total, 90,864 patients with EC were registered between 1973 and 2015. By applying the selection criteria, we identified 1,792 patients with pT1-3N0 thoracic esophageal cancer from 1998 to 2015 (Table 1). In total, 295 patients (16.5%) were aged 75 years or older, while 1497 patients (83.5%) were younger than 75 years. The patients in the older group were more likely to be female (23.4% vs. 16.5%; *P* = .005), be widowed (17.6% vs 6.3%; *P* < .001), have cancer located in the middle third of the esophagus (18.6% vs 15.5%; *P* = .049), and have localized disease (24.1% vs 16.6%; *P* = .005) than those in the younger group. Additionally, the proportions of black (1.0% vs 5.5%; *P* = .003) and pT1 (54.2% vs 64.5%; *P* = .003) patients in the older group were significantly lower than those in the younger group.

**Table 1:** Patients’ characteristics from the overall cohort and each age group.

	Older (≥75 years)	Younger (<75 years)	
	n = 295	n = 1497	*P* value
Year of diagnosis			
1998-2003	67 (22.7)	341 (22.8)	.981
2004-2009	141 (47.8)	707 (47.2)	
2010-2015	87 (29.5)	449 (30.0)	
Sex			
Male	226 (76.6)	1250 (83.5)	.005
Female	69 (23.4)	247 (16.5)	
Race			
White	274 (92.9)	1344 (89.8)	.003
Black	3 (1.0)	82 (5.5)	
Others	18 (6.1)	71 (4.7)	
Marital status			
Single (never married)	22 (7.5)	229 (15.3)	<.001
Married (including common law)/ unmarried or domestic partner	199 (67.5)	1029 (68.7)	
Separated/Divorced	22 (7.5)	145 (9.7)	
Widowed	52 (17.6)	94 (6.3)	
Tumor location			
Upper third of esophagus	3 (1.0)	50 (3.3)	.049
Middle third of esophagus	55 (18.6)	232 (15.5)	
Lower third of esophagus	237 (80.3)	1215 (81.2)	
Histology			
Squamous cell carcinoma	70 (23.7)	304 (20.3)	.186
Adenocarcinoma	225 (76.3)	1193 (79.7)	
pT classification^∗^			
T1	160 (54.2)	966 (64.5)	.003
T2	65 (22.0)	268 (17.9)	
T3	70 (23.7)	263 (17.6)	
eLNs			
	12.342 ± 0.5210	13.447 ± 0.2778	.098
<15	196 (66.4)	952 (63.6)	.352
≥15	99 (33.6)	545 (36.4)	
SEER historic stage			
Localized	221 (74.9)	1216 (81.2)	.005
Regional	71 (24.1)	249 (16.6)	
Distant	3 (1.0)	32 (2.1)	
Grade			
G1	39 (13.2)	247 (16.5)	.275
G2	142 (48.1)	726 (48.5)	
G3	114 (38.6)	524 (35.0)	

eLNs = examined lymph nodes, pT classification = pathologic T classification.

∗ The 8th edition of the TNM staging system was used as a reference in the study.

### 3.2 Impact of eLNs on survival

**Figure 1:**
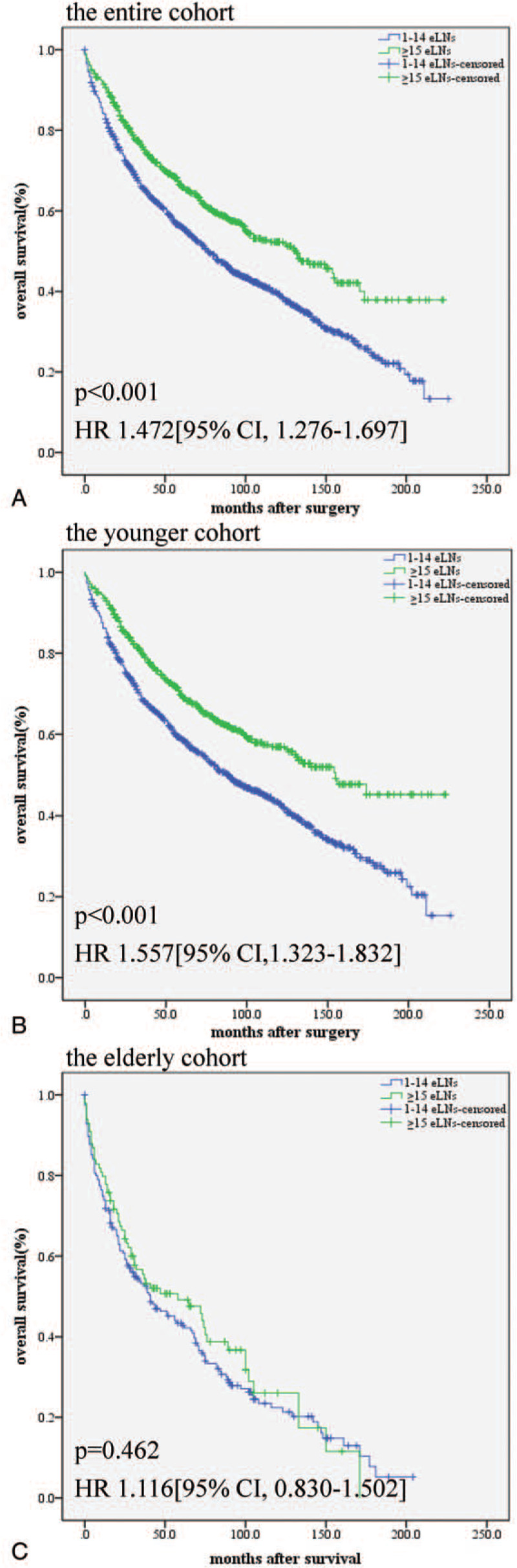
Postoperative overall survival curves according to the subgroups of eLNs (1-14 eLNs or ≥15 eLNs). (A) Overall survival of the entire cohort (all ages); (B) overall survival of the younger cohort (<75 years); and (C) overall survival of the older cohort (≥75 years).

The 5-year overall survival rates were 59.7% in the overall cohort, 56.1% in the patients who underwent esophagectomy with 1 to 14 eLNs, and 66.3% in the patients who underwent esophagectomy with ≥15 eLNs. The long-term overall survival in the patients with ≥15 ELNs was significantly superior to that in the patients with 1–14 ELNs (*P* < .001, Fig. 1A). We further performed an age-stratified overall survival analysis in each age group between the subgroups of eLNs. The 5-year overall survival rates in the younger patients were 58.6% in the patients with 1–14 eLNs and 69.5% in the patients with ≥15 eLNs (*P* < .001, Fig. 1B). However, the difference between the subgroups of eLNs disappeared in the elderly patients. The 5-year overall survival rates in the elderly patients were 43.4% in the patients with 1 TO 14 eLNs and 49.2% in the patients with ≥15 eLNs (*P* = .462, Fig. 1C).

**Figure 2:**
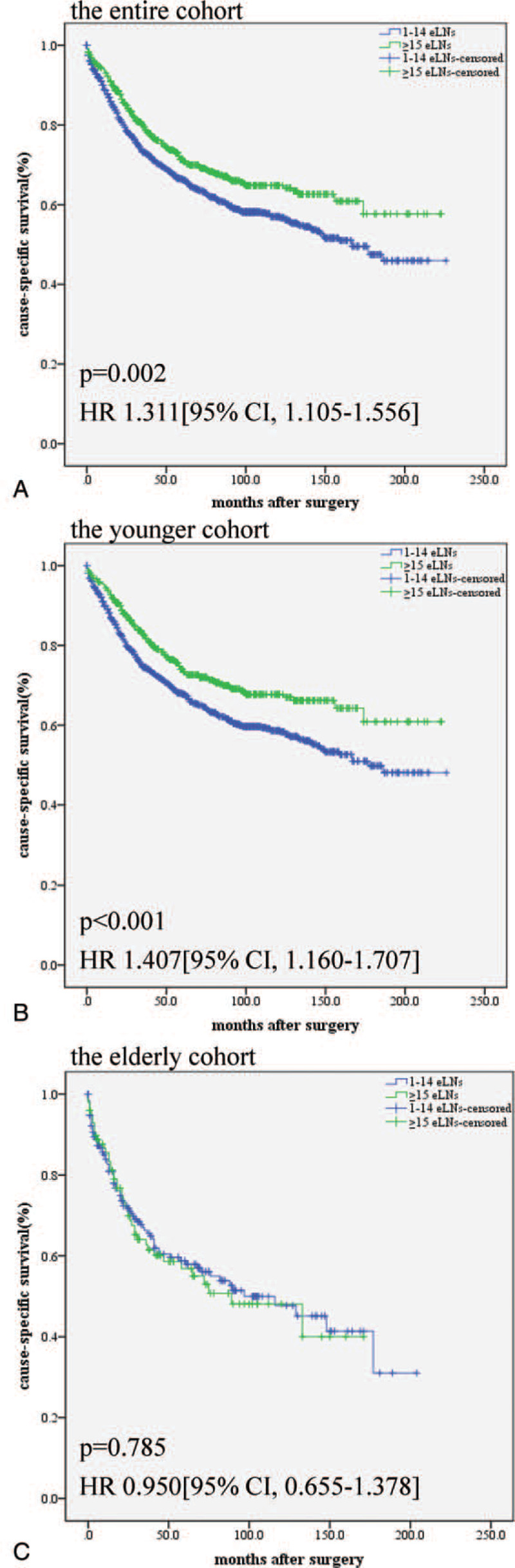
Postoperative cause-specific survival curves according to the subgroups of eLNs (1-14 eLNs or ≥15 eLNs). (A) Cause-specific survival of the entire cohort (all ages); (B) cause-specific survival of the younger cohort (<75 years); and (C) cause-specific survival of the older cohort (≥75 years).

Regarding cause-specific survival, we obtained results similar to the overall survival results. Kaplan-Meier plots of the subsets of patients in the different age groups with different numbers of eLNs are presented in Figure 2. As shown in Figure 2, the long-term cause-specific survival in the patients with ≥15 eLNs was significantly superior to that in the patients with 1 to 14 eLNs (*P* = .002, Fig. 2A), and the results of the subgroup analysis of the younger patients were similar (*P* < .001, Fig. 2B). However, the difference between the subgroups of eLNs disappeared in the elderly patients (*P* = .785, Fig. 2C).

### 3.3 Multivariate regression analyses of the impact of eLNs on survival

The multivariate analysis showed that the middle third of the esophagus, Esophageal squamous cell carcinoma, an advanced T classification (T3), fewer eLNs (1-14 eLNs), poor differentiation, and an advanced age (≥75 years) were significant independent unfavorable factors for overall survival (Table 2). To examine the overall survival effect of the number of eLNs stratified by age group, we classified the patients into the following 4 subgroups:

(1) younger patients with 1–14 eLNs;
(2) younger patients with ≥15 eLNs;
(3) elderly patients with 1–14 eLNs; and
(4) elderly patients with ≥15 eLNs.

**Table 2:** Multivariate analysis of survival.

	Overall survival		Cause-specific survival	
Characteristics	HRs (95% CI)	*P* value	HRs (95% CI)	*P* value
Tumor location				
Upper third of esophagus	1.375 (0.960–1.969)	.082	1.750 (1.169–2.619)	.007
Middle third of esophagus	1.378 (1.145–1.660)	.001	1.527 (1.220–1.910)	<.001
Lower third of esophagus	Baseline		Baseline	
Histology				
Squamous cell carcinoma	1.196 (1.007–1.421)	.041	1.247 (1.012–1.535)	.038
Adenocarcinoma	Baseline		Baseline	
pT classification				
T1	0.498 (0.277–0.897)	.020	0.430 (0.221–0.837)	.013
T2	0.848 (0.475–1.515)	.579	0.835 (0.434–1.606)	.589
T3	Baseline		Baseline	
eLNs				
<15	1.553 (1.344–1.793)	<.001	1.431 (1.203–1.702)	<.001
≥15	Baseline		Baseline	
Grade				
G1	0.743 (0.606–0.910)	.004	0.549 (0.417–0.724)	<.001
G2	0.818 (0.713–0.939)	.004	0.721 (0.609–0.852)	<.001
G3	Baseline		Baseline	
Age at diagnosis				
<75	0.551 (0.471–0.645)	<.001	0.676 (0.552–0.827)	<.001
≥75	Baseline		Baseline	
Stratification				
Younger patients with 1–14 eLNs	0.778 (0.596–1.014)	.064	0.860 (0.623–1.187)	.358
Younger patients with ≥15 eLNs	0.480 (0.360–0.639)	<.001	0.571 (0.405–0.805)	.001
Elderly patients with 1–14 eLNs	1.336 (0.991–1.800)	.057	1.173 (0.807–1.705)	.403
Elderly patients with ≥15 eLNs	Baseline		Baseline	

CI = confidence interval, eLNs = examined lymph nodes, HRs = subdistribution hazard ratio, pT classification = pathologic T classification. ^∗^ The 8th edition of the TNM staging system was used as a reference in the study.

Then, we calculated the hazard ratios of overall death in each patient group based on a multivariate Cox proportional model. Group D was used as a control group. As shown in Table 2, the hazard ratio of group C was similar to that of group D (1.336; 95% [CI], 0.991-1.800). The hazard ratio of group A (0.778; 95% CI, 0.596-1.014) was significantly higher than that of group B (0.480; 95% CI, 0.360-0.639). Regarding cause-specific survival, we obtained similar results (Table 2).

We further stratified the patients by the tumor location, histology, pT classification, and differentiation in the younger and older cohorts to analyze the association between eLNs and survival. As shown in Table 3, the differences remained significant in most subgroups, except for the middle third of the esophagus and G3 in the younger cohort (overall survival). No differences were observed in the elderly cohort (Table 4).

**Table 3:** Stratification analysis of eLNs on survival in the younger cohort.

	overall survival		cause-specific survival	
Characteristics	HRs (95% CI)	*P* value	HRs (95% CI)	*P* value
Tumor location				
Upper third of esophagus	5.564 (1.524–20.312)	.009	3.859 (1.054–14.134)	.041
Middle third of esophagus	1.242 (0.877–1.758)	.222	1.268 (0.846–1.903)	.251
Lower third of esophagus	1.701 (1.408–2.055)	<.001	1.522 (1.214–1.910)	<.001
Histology				
Squamous cell carcinoma	1.552 (1.135–2.122)	.006	1.543 (1.067–2.230)	.021
Adenocarcinoma	1.671 (1.377–2.026)	<.001	1.482 (1.177–1.866)	.001
pT classification^∗^				
T1	1.693 (1.341–2.137)	<.001	1.527 (1.133–2.059)	.005
T2	1.569 (1.097–2.245)	.014	1.268 (0.845–1.903)	.251
T3	1.592 (1.172–2.161)	.003	1.673 (1.199–2.332)	.002
Grade				
G1	4.467 (2.431–8.209)	<.001	3.287 (1.526–7.082)	.002
G2	1.663 (1.303–2.123)	<.001	1.569 (1.167–2.109)	.003
G3	1.263 (0.989–1.612)	.061	1.250 (0.944–1.656)	.119

CI = confidence interval, eLNs = examined lymph nodes, HRs = subdistribution hazard ratio, pT classification = pathologic T classification.

∗ The 8th edition of the TNM staging system was used as a reference in the study.

**Table 4:** Stratification analysis of eLNs on survival in the older cohort.

	overall survival		cause-specific survival	
Characteristics	HRs (95% CI)	*P* value	HRs (95% CI)	*P* value
Tumor location				
Upper third of esophagus	NA$	NA$	NA$	NA$
Middle third of esophagus	2.075 (1.051–4.096)	.035	1.680 (0.774–3.643)	.189
Lower third of esophagus	1.172 (0.826–1.663)	.373	1.018 (0.649–1.596)	.940
Histology				
Squamous cell carcinoma	1.126 (0.586–2.161)	.722	0.972 (0.462–2.045)	.940
Adenocarcinoma	1.361 (0.942–1.968)	.101	1.268 (0.787–2.042)	.329
pT classification^∗^				
T1	1.300 (0.803–2.106)	.286	1.224 (0.638–2.348)	.542
T2	1.459 (0.797–2.671)	.221	1.277 (0.599–2.721)	.526
T3	1.222 (0.692–2.158)	.489	1.053 (0.548–2.023)	.877
Grade				
G1	1.321 (0.456–3.822)	.608	0.566 (0.143–2.243)	.418
G2	1.295 (0.813–2.063)	.276	1.270 (0.696–2.317)	.436
G3	1.289 (0.804–2.068)	.292	1.216 (0.697–2.119)	.491

CI = confidence interval, eLNs = examined lymph nodes, HRs = subdistribution hazard ratio, NA = not applicable, pT classification = pathologic T classification.

∗ The 8th edition of the TNM staging system was used as a reference in the study. $ Upper third of esophagus was not assessed because the number is only 3(1.0%).

## 4 Discussion

Esophagectomy with lymphadenectomy remains the fundamental curative modality for esophageal cancer. Clinicians have explored the role of lymphadenectomy in improving survival rates. Although several studies have indicated that the number of eLNs during esophagectomy for esophageal cancer does not influence prognosis,^[7,15,17]^ the benefit of more extensive lymphadenectomy has been widely accepted by most surgeons. Currently, the AJCC staging system and the National Comprehensive Cancer Network recommend the dissection of at least 10 and 15 LNs, respectively, at the time of esophagectomy. Undoubtedly, a possible trade-off exists between the potential survival benefit and increased postoperative morbidity and mortality associated with more extensive lymphadenectomy. It is well known that the range of dissected LNs should be based on the probability of lymph node metastasis; thus, patients with the pN0 classification may benefit less from extensive lymphadenectomy. However, studies have demonstrated that elderly patients have a higher risk of postoperative morbidity and mortality than their younger counterparts,^[14]^ which may counteract the benefits of surgery. Therefore, the benefits of extensive lymphadenectomy remain to be determined and have not been defined in clinical practice in elderly patients with pN0 thoracic esophageal cancer.

The latest SEER database was used to perform a survival analysis of elderly patients (≥75 years) and their younger counterparts (<75 years). Our results demonstrate that the long-term survival of patients with ≥15 eLNs was significantly superior to that of patients with 1-14 eLNs in the overall and younger cohorts. However, the difference between the subgroups of eLNs disappeared in the elderly patients. Furthermore, multivariate analyses were performed to identify the association between eLNs and survival in patients stratified by several variables, such as age at diagnosis, tumor location, histology, pT classification and grade. The results showed that the differences remained significant in most subgroups in the younger cohort. Regarding the patients in the elderly cohort, no differences were observed in any subgroup.

Relapse in pN0 esophageal cancer patients remains problematic, and studies have shown that eLN counts exhibit prognostic significance in such patients.^[2,12,19,20]^ Lymph node micrometastasis (LNMM) and staging migration may account for this phenomenon. Accordingly, examining more LNs may eliminate micrometastatic lymph nodes, increase the likelihood of accurate staging and influence survival.^[18]^ First, the reported proportion of LNMM undetectable by conventional histopathology ranges from 14.2% to 85.7% in pN0 patients with esophageal cancer.^[5,6]^ However, the clinical significance remains controversial. Of the dozens of articles concerning LNMM in esophageal cancer, only 2 studies did not emphasize its prognostic value.^[1,13]^ Therefore, much evidence is available suggesting that LNMM is of clinical significance in patients with esophageal cancer. Second, the correct LN staging is crucial in evaluating the prognosis of patients with esophageal cancer. As the number of eLNs during surgery increases, the probability of missing a micrometastatic LN and the proportion of patients with higher-stage disease who are misclassified as having an early-stage cancer decrease. This resulting stage migration phenomenon may result in a poor prognosis associated with few eLNs. Third, it is possible that a high-volume center or an experienced surgeon tends to remove a greater number of LNs, resulting in better survival.

However, few studies investigated elderly patients with pN0 thoracic esophageal cancer. As a special cohort, elderly patients tend to have more comorbidities, a limited life expectancy, a worse general condition, and less tolerance for esophagectomy with lymphadenectomy compared with their younger counterparts.^[14,21]^ In clinical practice, it is often difficult to judge the indication for extensive lymphadenectomy in elderly patients as their decreased physiologic reserve can potentially place elderly patients at a higher risk of complications or mortality during esophagectomy; in these patients, additional extensive lymphadenectomy may not provide meaningful prolongation of life. To the best of our knowledge, no previous research focused on the impact of the eLN count on prognosis in elderly patients with thoracic pN0 EC. Our study demonstrates that examining a greater number of LNs did not appear to improve the outcomes in these elderly esophageal cancer patients. As data concerning local recurrence and distant metastases are missing in the SEER database, we calculated the cause-specific survival, which may be related to tumor recurrence and metastases. As shown in the results section, we obtained similar overall and cause-specific survival results, indicating that a less extensive lymphadenectomy is not associated with tumor recurrence and metastases in elderly patients.

However, the interpretation of our results is restricted by several limitations. First, given the lack of detailed provider information in the SEER database, we were unable to assess comorbidities, which frequently occur in elderly patients and may negatively affect survival. Second, we did not distinguish between different surgical approaches. Third, we were unable to link data regarding the surgical or hospital volume with the number of eLNs to assess whether this factor confounded the results. Finally, the argument regarding lymphadenectomy primarily focuses on the number and location of lymph nodes; however, we failed to assess the effect of the location of the resected LNs on survival. Despite these limitations, there are several advantages to using SEER data in this study. Specifically, the large number of patients and long-term follow-up duration enabled the reporting of overall and cause-specific survival and provided enough evidence to compare survival in patients with different numbers of eLNs. Our study provides important evidence suggesting that less extensive lymphadenectomy after esophagectomy, which is not associated with a worse prognosis, higher tumor recurrence or metastases, may be an acceptable treatment modality in elderly patients with pN0 thoracic esophageal cancer.

## 5 Conclusion

This study replicates the finding that the long-term survival of patients with extensive lymphadenectomy is significantly superior to that of patients with less extensive lymphadenectomy. However, less extensive lymphadenectomy may be an acceptable treatment modality in elderly patients with pN0 thoracic esophageal cancer.

## Author contributions

**Conceptualization:** Guoqing Zhang, Xiaofeng Guo, Lulu Yuan, Zhen Gao, Xiangnan Li.

**Data curation:** Guoqing Zhang, Xiaofeng Guo, Lulu Yuan, Xiangnan Li.

**Formal analysis:** Guoqing Zhang, Xiaofeng Guo, Lulu Yuan.

**Funding acquisition:** Guoqing Zhang, Xiaofeng Guo, Lulu Yuan, Jindong Li, Xiangnan Li.

**Investigation:** Guoqing Zhang, Xiaofeng Guo, Lulu Yuan, Zhen Gao, Xiangnan Li.

**Methodology:** Guoqing Zhang, Xiaofeng Guo, Lulu Yuan, Zhen Gao, Jindong Li, Xiangnan Li.

**Project administration:** Guoqing Zhang, Xiaofeng Guo, Lulu Yuan, Zhen Gao, Xiangnan Li.

**Resources:** Guoqing Zhang, Xiaofeng Guo, Lulu Yuan, Xiangnan Li.

**Software:** Guoqing Zhang, Xiaofeng Guo, Lulu Yuan.

**Supervision:** Jindong Li, Xiangnan Li.

**Validation:** Guoqing Zhang, Xiaofeng Guo, Lulu Yuan, Jindong Li, Xiangnan Li.

**Visualization:** Guoqing Zhang, Jindong Li.

**Writing – original draft:** Guoqing Zhang, Zhen Gao.

**Writing – review & editing:** Guoqing Zhang, Xiaofeng Guo, Lulu Yuan.

## Abbreviations

Abbreviations: AJCC = the American Joint Committee on Cancer, CI = confidence interval, eLNs = examined lymph nodes, LN = lymph node, pT classification = pathologic T classification, SEER = Surveillance, Epidemiology, and End Results.
